# Fourth Report to the General Assembly of Rhode Island

**Published:** 1857-11

**Authors:** 


*Fourth Report to the General Assembly of Rhode Island, relating to the Registry and Returns of Births, Marriages and Deaths in the State for the year ending December* 31*st*, 1856. *Prepared under the direction of John R. Bartlett, Secretary of State.*

This report was confided to Charles W. Parsons, M. D., of Providence, R. I., by the committee of the General Assembly. Dr. Parsons prepared the two preceding reports. All three attest the industry, devotion and intelligence which so eminently qualify the Doctor for such service. A few such men scattered over this country, empowered and encouraged by the legislatures of all our States, would amass an amount of valuable and endurable information that would in all future time be an honor to our age. Too little interest is now manifested on the subject of registration by State authorities. It is to be hoped that this will not be long tolerated by so large, talented and influential a body of men as the physicians of this country now are. They alone can appreciate the importance of registration, and as they are now organized more thoroughly all over America than they ever have been hitherto, we believe they may and before long will be heard to some effect in this matter. We should act perseveringly and together on those—alas! often too stupid—bodies called legislatures, until they are universally awakened upon this great topic.

